# Comparison of serum level of some trace elements and vitamin D between patients with premenstrual syndrome and normal controls: A cross-sectional study

**DOI:** 10.18502/ijrm.v17i9.5100

**Published:** 2019-09-22

**Authors:** Mahnoosh Fatemi, Maryam Allahdadian, Mehrnoosh Bahadorani

**Affiliations:** ^1^Department of Biology, Falavarjan Branch, Islamic Azad University Isfahan Iran.; ^2^Department of Nursing and Midwifery, Falavarjan Branch, Islamic Azad University Isfahan Iran.

**Keywords:** Premenstrual syndrome, Trace elements, Vitamin D

## Abstract

**Background:**

Premenstrual syndrome (PMS) is a common problem among women and is identified by reversal emotional, psychological, and physical signs during the luteal phase. These signs, however, lower down in the follicular phase. The cause of PMS isn't very well-known up to nowControl group and many researchers have suggested that mineral compounds and vitamins can inhibit these symptoms

**Objective:**

The objective of this study is to compare the serum level of some trace elements and vitamin D between normal controls and patients with PMS.

**Materials and Methods:**

300 female students (19–21 yr old) from Falavarjan County were randomly selected and asked to complete a standard questionnaire on PMS during three menstruation cycles. The students were divided into two groups: healthy persons (control) and PMS persons, and PMS was determined on the basis of the answers to the questionnaire. Thereafter, the serum concentrations of zinc, iron, calcium, magnesium, potassium, sodium, and Vitamin D3 were measured and compared between the two groups.

**Results:**

Our results showed that the PMS prevalence was about 41.5%. The level of vitamin D decreased in both the control and PMS groups, with a significantly lower range of vitamin D (p ≤ 0.05) in the PMS group. Other factors had no significant change between the two groups.

**Conclusion:**

Vitamin D deficiency was probably one of the most important causes of unpleasant symptoms of PMS between these students.

## 1 Introduction

Premenstrual syndrome (PMS) is one of the major health issue among women all over the world. This syndrome is determined with more than 150 physical, behavioral, and psychological characters, which begin during the luteal phase (7 to 14 days before ovulation) and end in the menstrual phase. Epidemiological studies have shown that about 80–90% of women have at least one of the PMS sings; however, in about 2.5–3% of women, the syndrome is so severe that it affects their personal activities and social communications. This disorder is called premenstrual dysphoric disorder (PMDD) (1).

In spite of a host of studies, pathophysiological causes of this syndrome remain unknown. PMS symptoms occur in the menstrual cycle, so they can be linked with hypothalamus-pituitary-gonad axis deficiencies. Some researchers have established that progesterone affects the secretion of some neurotransmitters such as serotonin, GABA, opioid, and catecholamine, and causes PMS signs to appear (2).

While others believed that a deficiency in adrenal hormone secretion and body electrolyte imbalance are responsible for these symptoms. Hormone-like and tranquilizing drugs and traditional medicines are used to treat some symptoms of PMS (1). Many women experimented and found that vitamins and trace elements (calcium, magnesium, iron) supplements can reduce PMS symptoms, which has been confirmed by some researches as well. Minerals and vitamins play important roles as cofactor and coenzyme in endocrine hormone synthesis such as in hypothalamus-pituitary-ovary axis (3), hence their deficiency might lead to similar medical signs as PMS symptoms in the luteal phase.

In this study, the percentage of PMS prevalence in a number of students from the city of Falavarjan was estimated, and the relationship of some serum trace elements as well as vitamin D in suffered and normal students was assessed.

## 2 Materials and Methods

This study was a cross-sectional study on 300 female students (aged 19-21-yr old, age of first menstrual period 13–14-yr old) from Falavarjan County between May and December 2016. They were assessed based on the PMS questionnaire. This questionnaire was based on an Iranian version of the premenstrual symptoms screening tool (PSST) (4), including 25 questions related to general health and gynecological condition. Students were asked to mark their signs and symptoms during the last three menstruation cycles, and in this way, having PMS was proven. Someone who suffered any endocrine disorder or was taking vitamin and mineral complements, corticosteroid antidepressants, and oral contraceptive pill were removed from the study.

Two wk after the menstrual period ended during the luteal phase, vein blood samples were taken following a fasting night. Serum samples were prepared (2500 rpm, 10 min), and then mineral parameters and vitamin D were assessed.

The serum concentration of vitamin D was measured by dihydroxy vitamin D diagnosis ELISA kit (from Bio Rex Fars Inc.). Its normal range was considered 30-70 ng/ml. The serum concentration of calcium and magnesium were calculated by Pars Azmoon kit using auto analyzer (Arsenazo method) and photometric (Xylidl blue method), respectively. The normal range of calcium and magnesium were 8.2–10.6 mg/dl and 1.9-2.5 mg/dl, respectively.

The serum concentration of iron and zinc were evaluated by photometric method (Iron assay kit, Abcam Company) and atomic absorption flame method (Chemtech CTA 3000 England kit, wavelength: 213.9 nm and a slit width of 0.7 nm), respectively. The normal range of iron was assumed 37–165 Ug/dl (for 12-25-yr old) and zinc serum concentration less than 70 Ug/dl was presumed as zinc deficiency.

The serum concentration of sodium and potassium were assessed by the flame photometric method. The normal range of sodium and potassium concentrations was 135-145 mEq/L and 3.5-5 mEq/L, respectively.

### 2.1 Ethical consideration

This study was conducted with the approval of the Ethics Committee of Falavarjan(No: IR.IAU.NAJAFABAD.REC.1397.017). All students were informed about the project and had given written consent before participating in the study.

### 2.2 Statistical Analysis

PMS percentage was calculated by descriptive statistics. Normal distribution data verification of serum factors was done by Kolmogorov-Smirnov test. The results are presented as means ± SEM. The significance of differences of all results was analyzed by independent *t-*test. Significance was considered as p ≤ 0.05.

## 3 Results

**Figure 1:**
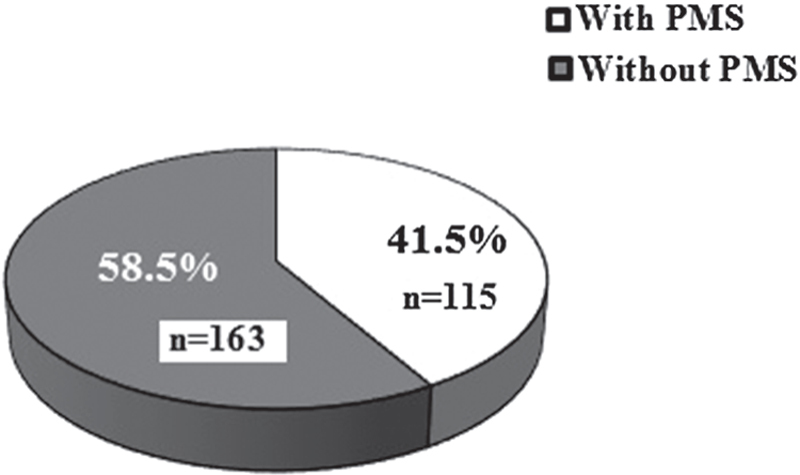
The prevalence of PMS in female students from Falavarjan County (n = 278).

**Figure 2:**
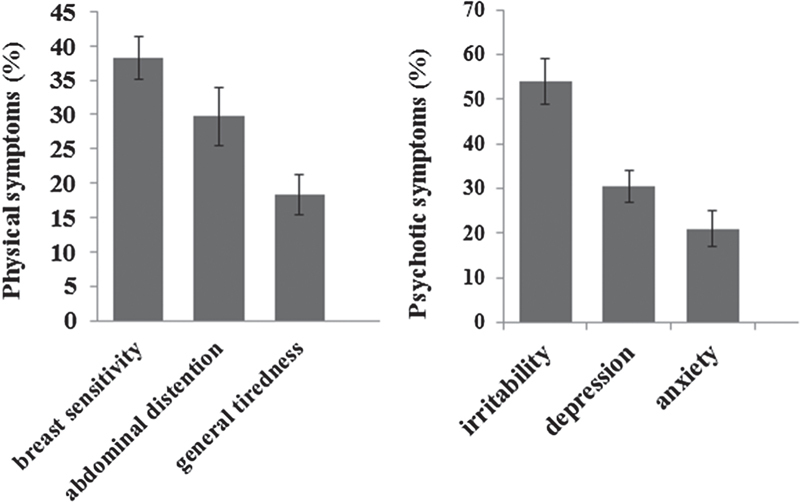
Distribution of some of the physical and psychological symptoms among female with PMS.

Two hundred and seventy eight cases out of three hundred samples were eligible for the analysis. Based on DSM-IV, the prevalence distribution of PMS was 41.52% (Figure 1). According to Figure 2, the highest percentage of psychotic symptoms in PMS groups were irritability (53.91%), depression (30.43%), and anxiety (21%), and the maximum percentage of physical signs were breast sensitivity (21.74%), abdominal distention (19.13%), and general tiredness (18%). The serum concentration of calcium, magnesium, zinc, iron, sodium/potassium ratio, and vitamin D of PMS group were less than the control group, but reveal no significant difference except for vitamin D (p ≤ 0.05). It should be noted that vitamin D deficiency was observed in both the PMS and control groups (198 out of 278 cases, 71.22%; Table I).

**Table I:** Serum levels of vitamin D and traces elements in the PMS and control groups

Factors	Control group	PMS group	P-value^*^
Calcium	9.79 ± 0.38	9.72 ± 0.43	0.265
Magnesium	2.10 ± 0.25	2.08 ± 0.21	0.672
Sodium/potassium	34.06 ± 2.31	33.67 ± 2.07	0.150
Zinc	84.41 ± 11.8	83.49 ± 11.58	0.521
Iron	85.76 ± 38.74	85.20 ± 25.08	0.857
Vitamin D	26.58 ± 6.74	22.44 ± 13.91	0.043^*^

Values are means ± SEM; the results were analyzed using Independent student t-test.

## 4 Discussion

In our study, the PMS prevalence was 41.5%, so our findings are almost in line with the previous researches in Iran. The PMS prevalence among the students of Shahid Beheshti of Tehran, the Medical Sciences of Uromia (5,6) and the Medical Sciences of Mashhad (7) were 47.07%, 39.4%, and 48.1%, respectively. Based on the meta-analysis and the systematic review of published papers during 1996-2011, on ISI, PubMed, and Scopus databases, the mean of the global PMS prevalence was reported as 47.8% (8). According to our results, irritability and breast sensitivity are the most abundant psychotic and physical signs, respectively. Even though our results differ from some previous studies, our findings were consistent with the most researches, as breast sensitivity, abdominal distention, headache, weight gain, fatigue, anxiety, depression, and anger were the most abundant physical and psychotic symptoms compared to the other signs (9,10). It is certain that the PMS sign percentage and intensity differences can be related to the diversity of diagnostic criteria, used methodology, climatic, cultural, and geographic differences, and even volunteer’s age and their lifestyle (1).

In regard to the probable relation of PMS signs to serum levels of essential elements, serum levels of calcium, magnesium, iron, zinc, potassium, and sodium were also compared between the two groups. The results showed a decrease in the level of these elements in the PMS group compared with the control group; of course, this reduction was not significant. Mira and colleagues investigated the plasma levels of magnesium and zinc and E and B6 vitamins in PMS cases and didn't observe any relation between the PMS signs and these serum parameters (11). Another study showed an insignificant reduction of magnesium and calcium in the PMS group (12). In contrast, some investigation confirmed the significant reduction of zinc level and total antioxidant capacity (13) in the PMS group and copper, cadmium, calcium, and magnesium reduction and ratio increase of magnesium to calcium and potassium to sodium in erythrocyte of PMS cases (14).

Although there are few contradictory documents indicating no relationship between serum vitamins and trace elements and PMS symptoms, many types of research showed that the consumption of this complement is effective in the management of unpleasant PMS symptoms. For instance, iron, magnesium, zinc, and potassium consumption by 1,968 healthy women during 10 years reduced the risk of PMS (15). The consumption of A, B, C, and E vitamins and magnesium and calcium (16) and the consumption of non-hem ferric and reduction of salt consumption (15) decreased PMS signs. Our study and other researches showed no relationship between serum elements and PMS. But why the consumption of essential elements did help PMS sign management? Although the levels of neurotransmitters and sexual hormones in PMS and non-PMS cases are similar, the oscillations of these factors from follicular to luteal phase are gradual in normal persons and sudden in PMS cases (17). Therefore, these sudden oscillations could be the reason for the increase in an instantaneous need for effective factors including essential elements and vitamins and a slight decrease of these factors should lead to unpleasant PMS symptoms. Studies showed that the serum level of essential elements and vitamins reduced in PMS cases but the reduction was insignificant. It is worth noting that even the minimum normal range of these factors should be considered in PMS cases and should rise to a higher degree.

Vitamin D reduction has been proven in many Iranian societies, both male and female (18), but a very few documents compare its levels in PMS and non-PMS times. A number of studies have shown a reduction of some PMS symptoms including anxiety, irritability, homesickness, and crying, following vitamin D consumption in PMS cases suffering vitamin D deficiency (19,20). In this study, the level of vitamin D in both normal and PMS groups decreased, but the PMS cases had a significantly lower range of vitamin D (p ≤0.05).

Some researchers have reported vitamin D reduction during the luteal phase in PMS women (19). In spite of these reports, the major circulating form of vitamin D (25 (OH) D) didn't show any significant difference between the control and PMS cases in other researches (21,22).

Vitamin D is a neurosteroid and its receptors are located in different parts of the brain, especially in the hippocampus. This vitamin and its receptor have been linked to several brain disorders, including cognitive decline, epilepsy, affective disorders, and schizophrenia (23).Vitamin D also has receptors on immune cells and as immunomodulator can inhibit inflammatory reaction during the menstrual cycle. Moreover, this vitamin prevents prostaglandins production by inhibition of cyclooxygenase 2 and NO synthesis (24,25). With regard to the two major factors of body pain that are pre-inflammatory cytokines and prostaglandins (25), vitamin D deficiency or defects in its receptors may cause mental and physical disorders in PMS cases.

Recently, several genetic mutations have been known in the vitamin D receptor (VDR). One of them, among many others, is the Fok1 single nucleotide polymorphism that has been linked to gynecological disorders as uterine leiomyoma and polycystic ovary (26,27). Hussain *et al.* according to a study on 340 female students from the Dubai Medical University reported that the frequency of F-containing genotypes between VDR Fok1 genotypes (FF, Ff, and ff) was significantly higher among the PMS group (56.8%) than the control group (44.1%). Moreover, the risk of susceptibility to depressive, PMS, and mood disorders was markedly increased in ff genotype (28,29). Due to the effect of VDR-Fok1 polymorphism on the mood and daily activity (30) in our research, this polymorphism has probably also played a role. However, widespread studies need to verify this claim.

## 5 Conclusion

Our results showed that the PMS prevalence was about 41.5% among a number of students from the city of Falavarjan. There were no significant differences in the serum levels of a number of trace elements in the PMS group compared with the health group. Although the vitamin D levels in both the groups were lower than the normal range, in the PMS group, it was significantly less than the health group. So in order to reduce the PMS symptoms, serum level of vitamin D should be at the highest level within the normal range.

## Conflict of Interest

The authors declare that there is no conflict of interests.
